# Polyspermy in birds: sperm numbers and embryo survival

**DOI:** 10.1098/rspb.2015.1682

**Published:** 2015-11-07

**Authors:** N. Hemmings, T. R. Birkhead

**Affiliations:** Department of Animal and Plant Sciences, University of Sheffield, Sheffield S102TN, UK

**Keywords:** embryo development, domestic fowl, fertilization, ovum activation, supernumerary sperm, zebra finch

## Abstract

Polyspermy is a major puzzle in reproductive biology. In some taxa, multiple sperm enter the ovum as part of the normal fertilization process, whereas in others, penetration of the ovum by more than one sperm is lethal. In birds, several sperm typically enter the germinal disc, yet only one fuses with the female pronucleus. It is unclear whether supernumerary sperm play an essential role in the avian fertilization process and, if they do, how females regulate the progression of sperm through the oviduct to ensure an appropriate number reach the ovum. Here, we show that when very few sperm penetrate the avian ovum, embryos are unlikely to survive beyond the earliest stages of development. We also show that when the number of inseminated sperm is limited, a greater proportion than expected reach and penetrate the ovum, indicating that females compensate for low sperm numbers in the oviduct. Our results suggest a functional role for supernumerary sperm in the processes of fertilization and early embryogenesis, providing an exciting expansion of our understanding of sperm function in birds.

## 1. Introduction

Males typically inseminate many more sperm than females need to fertilize their ova. Insemination of high sperm numbers increases a male's chance of success in sperm competition [1], but for females, high sperm numbers can be a problem; penetration by multiple sperm (polyspermy) is potentially destructive to the ovum [2]. Across virtually all internally fertilizing taxa, most sperm are destroyed or ejected by the female before getting close to the site of fertilization [3–5]. In birds, for example, of the hundreds of millions of sperm inseminated, only a few hundred reach the ovum [6], whereas in mammals, there is often only one [7,8].

The massive reduction in sperm numbers as they pass through the female reproductive tract between insemination and fertilization results from a series of anatomical and physiological ‘filters’ mediated by female selectivity [9,10]. These filters ensure that a non-random subset of sperm—the ‘fertilizing set’—reach the ovum [11,12]. What is less clear is how females strike the balance that ensures *sufficient* sperm reach the ovum, especially if they are inseminated by a sperm-depleted male (as is likely in lekking species [13]), or have limited opportunity to copulate close to ovulation (as in pelagic seabirds [14]). Ensuring that sufficient sperm are available for fertilization is particularly important for birds, in which several sperm typically enter the ovum in a process known as physiological polyspermy [15].

The discovery by Harper [16] that physiological polyspermy was a normal part of the avian fertilization process caused surprisingly little interest among biologists, both at the time and until very recently [17]. This is despite the fact that physiological polyspermy is so different from the situation in mammals, where penetration of the ovum by multiple sperm invariably results in embryo death (pathological polyspermy) [2].

Two studies of polyspermic fertilization in the domestic fowl *Gallus gallus domesticus* both concluded that while the maximum chance of fertilization success is achieved only when six or more additional (supernumerary) sperm enter the germinal disc, very low levels of fertilization success are still possible with just one or two penetrating sperm [18,19]. However, in neither study was the consequence of polyspermy for subsequent embryo survival considered. In addition, the method used to determine fertilization success in both these studies has been shown to overestimate infertility and underestimate early embryo death [20]. Whether supernumerary sperm have a functional role during the early stages of embryogenesis therefore remains in question.

Recently, *in vitro* studies of fertilization in Japanese quail *Coturnix japonica* demonstrated that the amount of avian sperm extract (containing ovum-activating proteins) required for normal post-fertilization development is greater than can be provided by a single sperm [21]. This suggests that under natural conditions, a minimum number of sperm must enter the ovum to ensure zygote formation and development.

Assuming physiological polyspermy to be an *essential* feature of avian reproduction, females with limited access to sperm may be expected to: (i) adjust the proportion of inseminated sperm they retain, and/or (ii) make it easier for retained sperm to reach the site of fertilization. In practical terms, either of these could be achieved by females filtering sperm less intensely and/or assisting the transport of sperm through the oviduct, because both strategies would result in a greater proportion of sperm reaching the ovum. Data from a study of artificially inseminated domestic fowl [5] are consistent with this hypothesis, but because the number of sperm inseminated—even in the smallest doses—was several orders of magnitude greater than what male fowl naturally inseminate [22], the biological significance of the results is questionable.

The aim of this study was to investigate how female birds respond to sperm limitation and how this influences sperm transport in the oviduct. Using two model species, the domestic fowl and zebra finch *Taeniopygia guttata*, we show that when fewer sperm are inseminated, a greater proportion reach and penetrate the region of the ovum where the germinal disc is located. We also show that penetration of the ovum by few sperm has little impact on the likelihood of fertilization but, if too few sperm penetrate, embryo survival is significantly reduced. Our results indicate that when inseminated sperm numbers are low, female birds compensate by allowing a greater proportion of sperm to reach the site of fertilization. This ensures that sufficient supernumerary sperm enter the germinal disc, so that both fertilization and development proceed normally. Our results also support the hypothesis that supernumerary sperm play an essential role in avian embryogenesis.

## 2. Methods

### (a) Zebra finches

The zebra finches used in this experiment were from a captive population of over 800 birds, maintained at the University of Sheffield since 1985. Sixteen female zebra finches were used, each producing clutches under both control and sperm-limited treatments. For the control treatment, pairs were allowed to copulate freely prior to the onset of egg laying. On the day of first oviposition, the male was placed behind a wire divider to prevent further physical interaction during the laying period. The sperm limitation treatment was achieved via artificial insemination; in preliminary artificial insemination trials (see the electronic supplementary material for further details), successful inseminations consistently resulted in extremely low sperm numbers reaching and penetrating the ovum. Although the underlying reason for this remains unknown, it provided a novel experimental technique by which we could assess fertilization and embryo survival when only tiny numbers of sperm enter the ovum. Pairs were kept in cages with a wire divider that separated the male and female physically to prevent copulations, but not visually or acoustically. Females were artificially inseminated on the day of first oviposition. Females under both treatments were habituated to the artificial insemination procedure via daily handling and mock inseminations carried out under licence throughout the entire experimental period (see the electronic supplementary material for further details).

Eggs were removed from nests daily and replaced with replica eggs to encourage clutch completion. Only the first four eggs per clutch were examined to avoid any confounding effects of sperm age [23,24]. Eggs were artificially incubated (Brinsea Octagon 20 Advance; 37°C and 60% RH) for either 24 h (to count sperm and holes in the perivitelline layer, and to assess fertilization success) or 14 days (the full incubation period, to assess embryo survival). Incubation duration (i.e. 24 h or 14 days) was alternated across eggs from each clutch to allow within-clutch comparisons of sperm number and embryo survival.

All eggs incubated for 24 h were examined using the techniques described by Birkhead *et al*. [20], to: (i) distinguish between fertilized and unfertilized ova, (ii) count the number of sperm trapped in the outer perivitelline layer (i.e. the number of sperm that reached the ovum but did not penetrate), and (iii) count the number of holes made by sperm that penetrated the inner perivitelline layer. Unhatched eggs from the 14 days incubation set were also examined as above, although in cases where development had advanced beyond approximately 3 days, sperm and hole counts were not possible. If a dead embryo was present in an unhatched egg, the developmental stage of the embryo was determined using Hamburger & Hamilton's [25] criteria. All eggs were examined blind with respect to sperm number treatment.

For the analysis of sperm numbers and fertilization success (eggs incubated for 24 h), the number of sperm and holes was counted for 95 eggs from 16 females (53 and 42 eggs from the control and sperm-limited treatments, respectively), including only eggs with one or more holes in the inner perivitelline layer. Eggs with no holes were excluded (19 and 107 from the control and sperm-limited treatments, respectively), because by definition [20], these were unfertilized—in all cases, germinal discs were checked for embryonic nuclei, and none were found.

For the analysis of embryo survival (fertile eggs incubated full-term), 57 fertilized eggs from 11 females (30 and 27 from the control and sperm-limited treatments, respectively) were incubated full-term and these were recorded as either hatched or unhatched. The other seven females from the original 16 (see above) did not produce a fertile egg that was incubated full-term in both treatments, so these females could not be included in this analysis. The effects of the number of sperm penetrating the ovum on fertilization success and embryo survival were analysed using generalized linear-mixed effects models (*glmer* function from the *lme4* package, R v. 3.1.2) with a binomial error distribution (owing to the binary nature of the response variables: fertilized/unfertilized, embryo survived/not survived), and female identity as a random effect (see the electronic supplementary material for further verification of these analyses).

### (b) Domestic fowl

Natural copulations in the zebra finch do not provide the ideal ‘control treatment’, because our comparison of relatively high and low sperm numbers is confounded by mode of insemination. We were also unable to quantify the absolute number of sperm inseminated. We therefore sought to validate our findings in another species—the domestic fowl—in which specific doses of both low and high sperm numbers could be artificially inseminated.

The domestic fowl used in this study were a Novogen Brown commercial layer strain (Tom Barron Ltd) maintained at The Roslin Institute, University of Edinburgh. Females (*n* = 21) were housed in groups of five or six in 1 × 2 m pens on wood shavings, at 21°C on a 14 h photoperiod. Semen was collected directly from eight cockerels by dorsoabdominal massage [26] and pooled. Sperm counts were performed using a haemocytometer in order to determine sperm concentration and the required semen dilutions were calculated for our sperm-limited and control doses (which contained approx. 10 000 and 10 000 000 sperm, respectively; appropriate dosages for the required sperm numbers reaching the egg were determined in preliminary trials). Semen was diluted accordingly (to equal total volumes) using Beltsville semen extender (Poultry and Pig Breeding Supplies Ltd.) and inseminated immediately. Females in two pens were artificially inseminated with the sperm-limited dose; females in the other two pens were artificially inseminated with control dose. The sperm numbers in semen samples from each dilution were counted retrospectively to confirm the dosage. It should be noted that semen extenders such as Beltsville, which are essentially buffered salt solutions based on the biochemical composition of semen [27], are designed to maintain the viability of sperm *in vitro*. It is possible that these solutions may also have positive effects on *in vivo* sperm function and fertility [28]. However, because all sperm samples were diluted prior to insemination in this study, any effects of the extender on sperm performance are likely to be consistent across treatments.

Eggs were collected from day two until day 7 following insemination. Eggs laid later than day 7 were not considered in order to avoid confounding effects of sperm age [23]. After 21 days (to allow for complete sperm depletion), the inseminations were repeated, but doses were switched across pens, so that each female received both sperm number treatments.

Eggs were collected each day; three eggs per dose per day were reserved to assess fertilization success and count sperm/holes. Eggs were examined as described for zebra finch, with the exception that, owing to the relatively large size of domestic fowl ova, counts were restricted to two 1 cm^2^ samples of perivitelline layer, one from the animal pole (above the germinal disc) and one from the vegetal pole. In total, 45 freshly examined eggs from 21 females (32 and 12 eggs from the high and low sperm number doses, respectively) were found to have one or more holes in the 1 cm^2^ area of perivitelline layer above the germinal disc. Eggs without any holes were excluded from the analysis (0 and 19 eggs from the high and low sperm number doses, respectively). No sperm were found in sperm-limited eggs laid more than 4 days after insemination, therefore, analyses of sperm, and hole numbers were restricted to this period. However, control eggs were found to have high sperm numbers throughout the 7 day focal period, indicating that the fertile period of control females was longer than that examined.

All other eggs were transferred to incubators (Bristol incubators S-30; 37.5°C and 60% RH) and incubated full-term (21 days) to assess embryo survival. In total, 82 incubated eggs were fertilized, but these were largely from the high sperm dose; 97% (72 out of 74) eggs from the high sperm dose were fertilized, compared with just 21% (10 out of 47) from the low sperm dose. Unhatched eggs were examined, and data analysed as described for the zebra finches above.

## 3. Results

### (a) Sperm compensation

**Figure 1.**
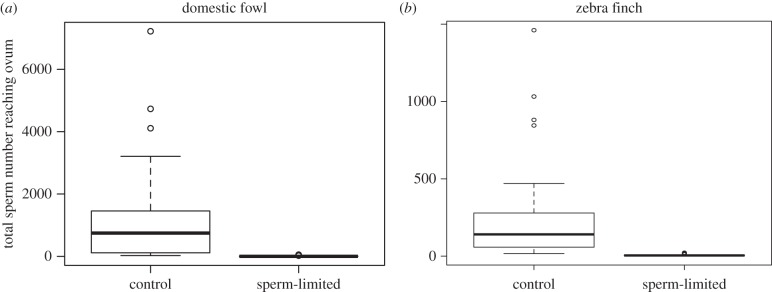
Tukey box plots show the effect of sperm limitation on absolute sperm numbers reaching ova in (*a*) domestic fowl (*n* = 17 eggs in each group, *z* = −50.98, *p* < 0.0001) and (*b*) zebra finches (*n* = 53 control and 42 sperm-limited eggs, *z* = −58.11, *p* < 0.0001).

**Figure 2.**
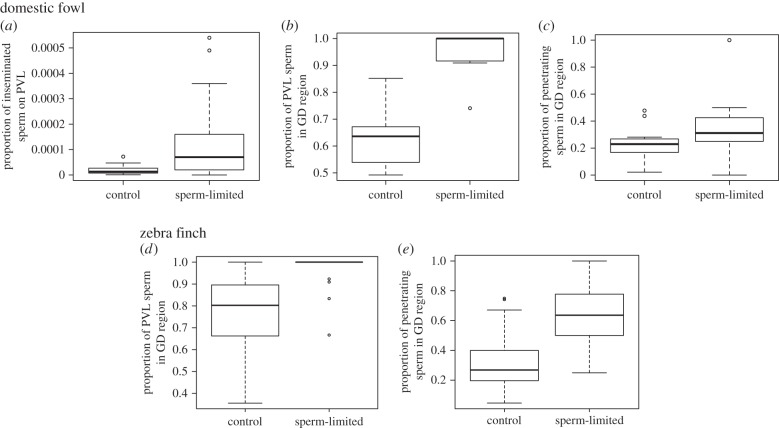
Tukey box plots show the proportion of (*a*) inseminated sperm that reached the ova during the first 4 days of egg-laying in the domestic fowl, following control and sperm-limited inseminations (*n* = 17 eggs in each group, mean proportion of sperm reaching ova = 0.13 × 10^−3^ (L-95% confidence interval (CI) = 0.08 × 10^−3^, U-95% CI = 0.18 × 10^−3^) and 1.19 × 10^−3^ (L-95% CI = 0.50 × 10^−3^, U-95% CI = 1.88 × 10^−3^), based on 10 000 000 and 10 000 inseminated sperm, respectively; *z* = 27.89; *p* < 0.0001); PVL, perivitelline layer; (*b,d*) sperm associated with the ovum that were in the germinal disc (GD) region in the domestic fowl (*n* = 17 control and 13 sperm-limited eggs; *z* = 31.54; *p* < 0.0001) and zebra finch (*n* = 53 control and 42 sperm-limited eggs; *z* = 6.99; *p* < 0.0001), respectively; (*c,e*) sperm associated with the germinal disc region that penetrated the ovum in the domestic fowl (*n* = 17 control and 13 sperm-limited eggs; *z* = 4.052; *p* < 0.0001) and zebra finch (*n* = 53 control and 42 sperm-limited eggs; *z* = 8.893; *p* < 0.0001), respectively.

Sperm limitation was highly effective in reducing the absolute number of sperm that reached ova in both the domestic fowl and zebra finch, compared with control treatments (figure 1). Despite this, the *proportion* of inseminated sperm that reached and penetrated ova from the sperm-limited treatment was significantly greater than it was for ova from control treatments (*p* < 0.0001; figure 2*a*). Specifically, in domestic fowl, the proportion of sperm found on ova in the first 4 days of the laying sequence (the maximum fertile period for sperm-limited inseminations) was approximately 14 times greater following sperm-limited inseminations than control inseminations. The rate at which sperm numbers declined across successive ova in the laying sequence did not differ between the control and sperm-limited groups (electronic supplementary material, figure S1). This indicates that rates of sperm release from storage were similar for both treatments, because the number of sperm on the perivitelline layer correlates strongly with the number in storage [29]; but see Discussion.

Compared with control ova, a greater proportion of perivitelline-bound sperm were located in the germinal disc region of sperm-limited ova than anywhere else on the perivitelline layer (*p* < 0.0001; figure 2*b*). Similarly, the proportion of sperm within the germinal disc region, that penetrated the perivitelline layer, was also greater in sperm-limited ova (*p* < 0.0001; figure 2*c*).

In the zebra finch, the absolute number of sperm inseminated was not quantifiable owing to the nature of our protocols (see Methods). However, as in the domestic fowl, a greater proportion of perivitelline-bound sperm were located in the germinal disc region of sperm-limited ova compared with control ova (*p* < 0.0001; figure 2*d*), and a significantly greater proportion of sperm in that region also penetrated the perivitelline layer (*p* < 0.0001; figure 2*e*).

### (b) Functional polyspermy

In the domestic fowl, fertilization success (confirmed by the detection of post-fertilization cell division) was significantly lower in sperm-limited ova than in control ova (8 out of 12 (77%) and 31 out of 32 (97%), respectively; *z* = −2.31; *p* = 0.021). This difference was driven by a threshold effect: ova penetrated by fewer than three sperm (*n* = 4) were unfertilized, whereas all other sperm-limited ova (*n* = 8) that had been penetrated by 3–17 (median = 4) sperm were fertilized. In control ova, the single unfertilized ovum had been penetrated by one sperm only; the remaining fertilized ova were penetrated by 4–868 (median = 82) sperm.

In the zebra finch, there was no difference in fertilization success between treatments: all sperm-limited ova penetrated by at least one sperm (*n* = 45) were fertilized, as were 52 out of 53 (98%) control ova (*z* = 0.002; *p* = 0.998), in which sperm penetration numbers ranged from 4 to 388. The high sperm penetration number for control ova in both species is consistent with numbers previously reported for a range of bird species [19,30].

Eggs fertilized under sperm limitation, however, were significantly less likely to hatch in both species. In the domestic fowl, none of 10 embryos produced under sperm limitation survived longer than 48 h, whereas 60 out of 72 (83%) embryos from control ova survived to hatch. In all cases of early embryo mortality, across both treatments, the number of sperm that had entered the germinal disc region at the time of fertilization was consistently low (two to five sperm only), suggesting a link between low supernumerary sperm numbers and early embryo death.

Similarly in the zebra finch, the sperm limitation treatment resulted in significantly lower embryo survival (*z* = −4.221; *p* < 0.001), with only 3 out of 27 (11%) embryos surviving to hatch. Of those embryos that died, 92% did so within 48 h of fertilization. By contrast, 21 out of 30 (60%) control embryos survived to hatch.

## 4. Discussion

We have shown that when female birds are inseminated with low sperm numbers, a greater number of sperm than expected reach and penetrate ova. Under our sperm limitation treatment in domestic fowl (10 000 sperm inseminated), the proportion of sperm that progressed to the site of fertilization was more than an order of magnitude greater than expected, based on the control treatment (10 000 000 sperm inseminated). This suggests that females regulate the number of sperm that reach ova, minimizing the risk of infertility when the number of inseminated sperm is low. We have also demonstrated that while only a very small number of sperm are necessary for fertilization (one in the zebra finch and three in the domestic fowl), larger numbers of sperm may be required for successful embryo development. Together, our results indicate that within the reproductive tract of female birds, low sperm numbers can be compensated for by enhanced progression to the ovum, ultimately ensuring that sufficient sperm are available for polyspermic fertilization and early embryogenesis.

The decline in perivitelline sperm numbers on consecutive ova was similar in both the control and sperm-limited treatments (electronic supplementary material, figure S1). Because perivitelline sperm numbers are known to correlate strongly with the number of sperm in storage [29], this suggests that rates of sperm loss from storage were similar for both treatments. If this is the case, then our results suggest that under sperm limitation, a greater proportion of inseminated sperm are able to enter storage. There are two ways in which females could regulate sperm numbers entering storage: by (i) passively allowing a fixed maximum number of sperm to reach the ovum, regardless of insemination dose; or (ii) actively adjusting the intensity of sperm selection prior to storage (either in the vagina or at the entrance to sperm storage tubules), in relation to sperm availability.

While the passive hypothesis (i) appears the more parsimonious, its validity is weakened by the fact that perivitelline sperm numbers are known to rise to a biologically unrealistic level with increasing insemination dose in domestic fowl [19]. The possibility that females actively control the rate of sperm acceptance into storage therefore warrants further investigation, particularly because evidence from other taxa suggests that, at least at the interspecific level, female selectivity for sperm fertilization success varies with sperm availability (e.g. Levitan 2002).

We also cannot rule out the possibility that under sperm limitation, the proportional relationship between stored and perivitelline sperm numbers breaks down (this has not been explicitly tested). It is therefore possible that females achieve sperm compensation via post-storage mechanisms such as enhanced sperm release from storage or assisted transport in the upper oviduct.

Whatever the underlying mechanism, allowing a greater proportion of sperm to reach and penetrate the ovum increases the chance that several sperm will enter the germinal disc—a situation we show to be essential for successful embryo development. However, as more sperm are allowed to reach the ovum, the chance of penetration by poor-quality sperm also increases. While this cannot be the sole explanation for the observed reduction of embryo survival in sperm-limited ova (very low sperm numbers were also associated with early embryo mortality in control ova), it may intensify the negative effects of low sperm numbers, resulting in a twofold impact on embryo survival.

The idea that polyspermy improves the chance of normal embryo development in birds is consistent with recent *in vitro* findings by Mizushima *et al*. [21]. Together, our studies support the hypothesis that supernumerary sperm have a functional role in early avian embryogenesis. This represents a fundamental expansion in our understanding of the function of sperm in fertilization, and goes some way to resolving long-standing questions regarding the biological significance of physiological polyspermy in birds [16,17].

The precise number of sperm required for embryo development remains in question. Mizushima *et al*. [21] showed that full ovum activation *in vitro* requires avian sperm extract, or its components: phospholipase C-zeta, aconitate hydratase and citrate synthase. These factors are thought to trigger both immediate and long-term Ca^2+^ oscillations that are essential for the progression of cell cycles during early embryogenesis. It is entirely feasible that any number of additional, as yet unknown sperm or semen factors may also be essential for the processes of fertilization and developmental initiation. However, while the volume of sperm extract required for successful development in Mizushima *et al*.'s [21] study came from the equivalent of approximately 200 sperm, our data indicate that the number of supernumerary sperm necessary for avian embryo development *in vivo* is far fewer. In addition, the exact number apparently differs between the two species we have studied, potentially increasing with ovum size.

If, as we suggest, poor sperm quality exacerbates the effect of low sperm numbers, the large number required *in vitro* may be the result of a complete lack of sperm selection prior to fertilization; Mizushima *et al*. [21] took sperm at random from ejaculated semen, which may have led to the use of non-functional sperm [31]. In addition, both the timing and location of supernumerary sperm entry into the germinal disc are likely to be crucial; in amphibians, for example, polyspermic ova are penetrated successively at different points on the ovum surface, and this has knock-on effects for the propagation of activating Ca^2+^ waves across the ovum [32,33]. Polyspermic fertilization may therefore be much more efficient under natural conditions than that which has so far been achieved *in vitro*.

An important future objective is to identify the mechanism by which polyspermic fertilization is regulated in birds, especially given the sometimes huge numbers (tens of thousands) of sperm that encounter the ovum [30]. The requirement for high sperm numbers at the site of fertilization in birds may be explained by the brief 15 min ‘fertilizable lifespan’ of avian ova [6]; to ensure sufficient sperm are present at precisely the right time, females release sperm from the storage tubules just prior to ovulation in response to hormonal cues [34], ensuring that numerous sperm populate the infundibulum prior to the release of the ovum. This differs considerably from the situation in mammals, where ova typically remain fertilizable for about 24 h in the fallopian tube [35], allowing greater scope for the female to regulate sperm numbers and achieve monospermy without the risk of infertility.

In conclusion, we have demonstrated that polyspermic fertilization increases the chance of embryo development in birds, and females appear to compensate for low sperm numbers by allowing a greater proportion to reach the ovum. Our findings, in combination with other recent work, provide an exciting new perspective on the processes underlying fertilization and embryogenesis in birds.

## Supplementary Material

Figure S1; Artificial insemination in zebra finches: Methods; Verification of glmer models using Bayesian generalised linear models

## Data accessibility

The following datasets relevant to this study are available from the Dryad digital repository (http://dx.doi.org/10.5061/dryad.9fg8m): (i) sperm numbers experimental data; (ii) fertilization success comparisons; and (iii) embryo survival comparisons.
