# Supplementary material for: Polyspermy in birds: sperm numbers and embryo survival
Source: Proc Biol Sci. 2015 Nov 7;282(1818):20151682. doi: 10.1098/rspb.2015.1682 (PMC4650155; doi:10.1098/rspb.2015.1682)
Supplement: Figure S1; Artificial insemination in zebra finches: Methods; Verification of glmer models using Bayesian generalised linear models [file rspb20151682supp1.pdf]

ESM 1 – Figure S1

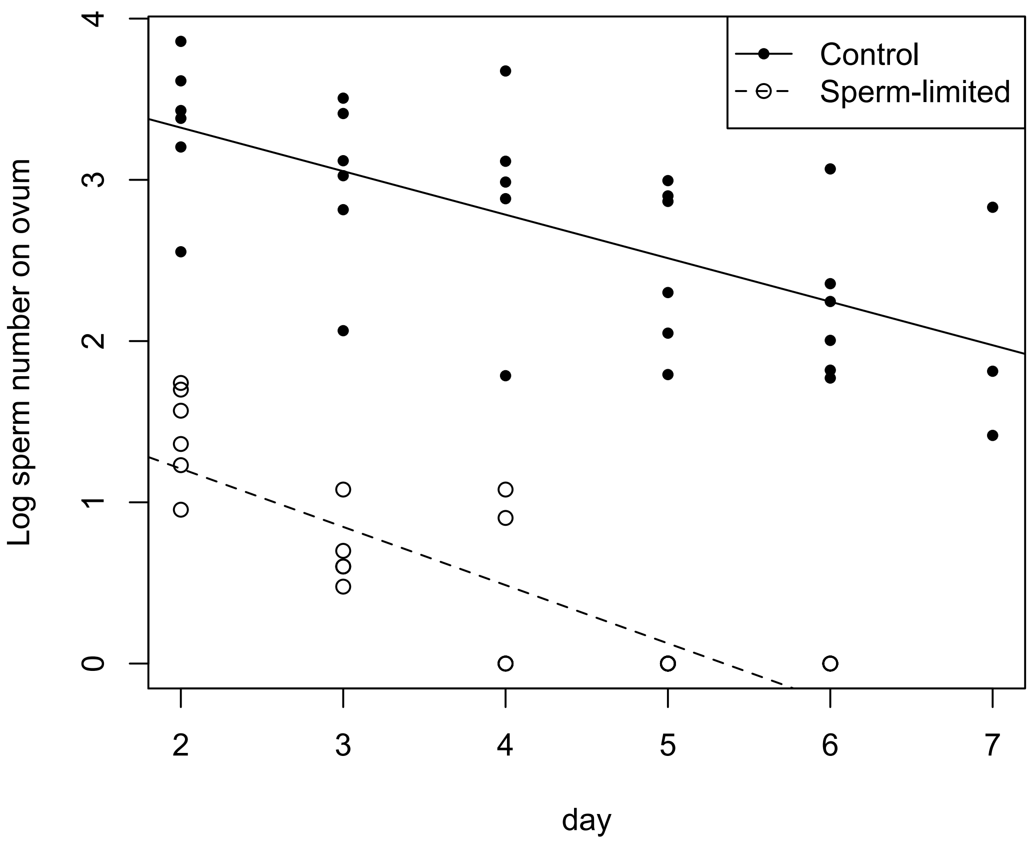

Figure S1. The decline in sperm numbers found on the perivitelline layer of ova following control (y = -0.27 + 0.06x; $R^2 = 0.42$; $F_{(1, 30)} = 23.56$; $p < 0.0001$) and sperm-limited (y = -0.36 + 0.04x; $R^2 = 0.72$; $F_{(1,29)} = 76.39$; $p < 0.0001$) inseminations, over the course of the laying sequence in domestic fowl. There was no significant interaction between the effects of insemination mode (control versus sperm-limited) and time ($F_{(1,59)} = 1.603$; $p = 0.21$) on sperm numbers, indicating that the rate of sperm loss did not differ significantly between control and sperm-limited trials.

ESM 2: Artificial Insemination in Zebra Finches: Methods

In preliminary trials of artificial insemination in the zebra finch (carried out under license from the UK Home Office and approved by local ethical review), we noted that successful inseminations consistently resulted in extremely low sperm numbers reaching and penetrating the ovum (as shown by the number of sperm and sperm penetration holes in the perivitelline layer; Wishart 1987). We do not know why these inseminations resulted in such low numbers of sperm reaching the ovum, but the most likely explanation is that sperm were deposited into the female at a location more external than they would be under natural insemination, limiting their ability to enter the reproductive tract. This method of "artificial insemination" has proved a useful tool for limiting the number of sperm reaching the ova (see article).

To encourage females under the 'low' treatment to lay a 'typical' clutch (i.e. 5-6 eggs, laid on consecutive days approximately one week after pair formation), male-female pairs were maintained in paired cages (single cage dimensions 0.6 x 0.5 x 0.4m) separated by a wire divider extending into a double nest-box. This allowed the pair to interact visually and acoustically but prevented copulations. Female zebra finches will build a nest and lay eggs normally under these conditions (NH, pers. obs.). In the control treatment, male-female pairs were kept in the same cages as used in the sperm-limited treatment, but without a wire divider. All birds were provided with food and water *ad libitum*, supplemented with boiled egg and lettuce and maintained at 20°C on a 12h photoperiod.

All females included in the experiment (under both control and sperm-limited treatments) were habituated to the “artificial insemination” procedure via daily handling and mock inseminations carried out under license. A mock insemination involved placing 5 µl of sterile water onto the female’s cloaca. For the ‘low’ treatment, habituated females were artificially inseminated (under license) between 1400-1600h on the day their first egg was laid, with 5µl semen taken directly from the seminal glomerus of dissected males. Based on sperm concentration data from a previous study on the same zebra finch population (Bennison *et al.* 2015), we estimated the approximate number of sperm in 5µl to be 0.675 x 10^6, which is slightly but not remarkably fewer than the number in a natural ejaculate (5.29 x 10^6 ± 1.56 x 10^6 for rested males, decreasing to 0.166 x 10^6 ± 0.08 x 10^6 in unrested males; Birkhead *et al.* 1995). Artificial insemination was identical in procedure to the mock inseminations, except that fresh semen instead of sterile water was placed on the cloaca. The female was held until the semen had disappeared into the cloaca (usually within 30s) and then returned to the cage. Females under the control treatment received a mock insemination at the same time.

References

Bennison, C., Hemmings, N. Slate, J. & Birkhead, T.R. 2015. Long sperm fertilise more eggs in a bird. *Proc. Roy. Soc. Lond. B.* **282**: 20141897

Birkhead, T.R. & Fletcher, F. 1995. Male phenotype and ejaculate quality in the zebra finch Taeniopygia guttata. *Proc. Roy. Soc. Lond. B.* **262**: 329-334.

Wishart, G.J. 1987. Regulation of the length of the fertile period in the domestic fowl by numbers of oviducal sperm, as reflected by those trapped in laid eggs. *J Reprod Fertil* **80**: 493-498

ESM 3: Verification of *glmer* models using Bayesian generalised linear models

Bayesian generalised linear models with Markov chain Monte Carlo (MCMC) estimation in *MCMCglmm*, R Version 3.1.2 (Hadfield 2010) were used to verify the *glmer* results concerning the effect of the sperm limitation treatment on fertility and embryo survival (reported in the main text). Due to a lack of variation in the data, the effect of sperm limitation on fertility in the zebra finch, and embryo survival in the domestic fowl, was not reanalysed. Results of the other *glmer* analyses were supported by the results of the *MCMCglmm* analyses: sperm limitation negatively influenced (a) embryo survival in the zebra finch (PDM = -0.38 x 10^3, L-95% CI = -0.73 x 10^3, U-95% CI = -0.11 x 10^3, pMCMC $<$ 0.001), and (b) fertility in the domestic fowl (PDM = -0.20 x 10^3, L-95% CI = -0.38 x 10^3, U-95% CI = 0.03 x 10^3, pMCMC = 0.008).

Reference

Hadfield, J.D. 2010. MCMC Methods for multi-response generalised linear mixed models: The MCMCglmm R package. *J Stat Softw* **33**: 1-22
